# Like a Complete Unknown: An Audit of the Quality of the Referrals to the Cancer of Unknown Primary Clinic at a Tertiary Care Centre

**DOI:** 10.3390/clinpract15070122

**Published:** 2025-06-26

**Authors:** Ian Hirsch, Jonah Teich, Khaled Abdulalem, Samuel D. Saibil

**Affiliations:** 1 Princess Margaret Cancer Centre, University Health Network, Toronto, ON M5G 2C4, Canada; 2 Department of Medicine, University of Toronto, Toronto, ON M5S 1A1, Canada

**Keywords:** carcinoma of unknown primary, quality improvement, knowledge-to-action cycle

## Abstract

Background: Carcinoma of Unknown Primary (CUP) constitutes approximately 3% of all advanced cancer cases globally, posing a distinct and complex medical challenge due to its metastatic nature, with no identifiable primary tumour site despite comprehensive investigations. Aim: This study aimed to assess the quality of referrals to the Cancer of Unknown Primary Clinic at the Princess Margaret Cancer Centre (PMCC) by conducting a retrospective audit of initial referrals between January 2022 and March 2023. Methods: The adequacy of referrals was evaluated based on adherence to NICE guidelines, focusing on essential diagnostic investigations such as comprehensive history, physical examination, CT scans, and pathological assessment with immunohistochemistry. Our cohort consisted of 97 patients with a median age of 66 years. Results: The results indicated that only 55% of referrals met the criteria for adequacy, with significant deficiencies in computed tomography (CT) scans and immunohistochemistry (IHC). Notably, the adequacy of referrals varied by specialty, with the lowest rates in emergency medicine and family medicine, and the highest rates in medical oncology, gastroenterology, and neurosurgery. Conclusions: These findings underscore the need for improved standardization and education to enhance referral quality, ensuring that patients with CUP receive appropriate and timely care. This study marks the initial phase of the Knowledge-to-Action cycle, highlighting areas for quality improvement in the referral process to the CUP clinic.

## 1. Introduction

Carcinoma of Unknown Primary (CUP) constitutes approximately 3% of all advanced cancer cases globally [1]. The diagnosis and treatment of CUP presents a distinct and complex medical challenge. CUP is defined by the presence of metastatic deposits with no identifiable primary tumour site despite comprehensive investigations (including advanced imaging and biopsies). Affecting a diverse demographic with an average diagnosis age between 60 and 75 years, CUP shows no significant skew towards any gender [2]. Its manifestation ranges from isolated metastases to extensive disease, affecting organs such as lymph nodes, bones, liver, and lungs, with metastatic adenocarcinoma being the most common presentation. Presentations can be subclassified as “favourable prognosis”, accounting for 20% of cases or “unfavourable prognosis”, accounting for the remaining 80%. [1] This classification is based on both the extent and the nature of the tumours, and in some cases the sex of the patient. Despite this range of presentations, the prognosis of CUP is generally poor, and the median overall survival is less than one year with treatment for patients with poor risk disease [3,4,5]. Timely and effective assessment by appropriate oncology specialists is therefore imperative to quickly initiate proper care and ensure that all appropriate assessment has been performed. Treatment of CUP depends on the diagnosis and classification of the disease. Those with favourable prognoses are generally managed similarly to patients with known primary site tumours matching the suspected origin of the CUP [6]. Those with unfavourable prognoses are generally managed with dual-agent platinum-based chemotherapy. Some recent studies suggest that a subset of CUP tumours may be particularly susceptible to immune checkpoint inhibitors, and that up to one third of CUP tumours may have genetic alterations for which targeted therapies exist [6,7,8,9,10]. Despite these advancements in therapeutic options, timely diagnosis remains imperative in the treatment of CUP.

The management of CUP demands a rigorous diagnostic process in line with established guidelines, such as the guidelines from the National Institute for Health and Care Excellence (NICE, UK) for Metastatic Malignant Disease of Unknown Primary Origin in Adults [6]. These guidelines offer healthcare professionals a comprehensive framework designed to enhance patient quality of life and inform treatment choices in CUP cases. The recommended diagnostic approach includes detailed history-taking, physical examination, various blood tests, imaging, and biopsy complemented by immunohistochemistry [8]. These recommended diagnostic tests are generally consistent across the guidelines published by various health agencies including, in addition to NICE, the National Institutes of Health (NIH) and the European Society for Medical Oncology (ESMO) [6,9]. Of note, ESMO recommends a broader selection of tumour markers for screening depending on the suspected site of origin [6]. Some recent guidelines have additionally started recommending gene expression profiling and next-generation sequencing (NGS) to aid in identifying the site of origin and guiding therapy [9,10]. A significant emphasis is placed on the formation of specialized CUP teams within hospitals, advocating for a collaborative approach to patient care. Establishing specialized teams for CUP in healthcare environments where resources are scarce or access to cancer care is uneven remains a significant hurdle [11]. Given these systemic limitations, it is imperative that only appropriate patients are referred to the CUP clinic, to ensure that patients with actual CUP are managed by disease specialists [12,13]. Unfortunately, it has been our experience that many of the referrals received at the CUP Clinic at the Princess Margaret Cancer Centre (PMCC), a tertiary care referral hospital, do not require the specialized knowledge of the CUP Clinic. To validate this hypothesis, we initiated a retrospective audit of the initial referrals received by the CUP Clinic at the PMCC between January 2022 to March 2023 as part of a Quality Improvement (QI) initiative. This analysis serves as the inaugural phase in the Knowledge-to-Action cycle [14], aiming to identify the extent of the problem and to eventually enhance the efficiency and efficacy of the referral process to the CUP Clinic to ensure that patients with CUP receive appropriate and timely care. This manuscript reports only the preliminary results of this analysis, as the other components of the Knowledge-to-Action cycle are ongoing.

## 2. Materials and Methods

A retrospective audit of initial referrals received at the CUP Clinic at the PMCC, Toronto between January 2022 to March 2023 was conducted. Our objective was to assess the adequacy of these referrals in accordance with the NICE guidelines [6]. Specifically, the focus was on assessing the number of recommended investigations for the initial diagnostic phase for patients with metastatic malignancy of unknown origin (MUO) that were performed prior to the referral being sent to the CUP Clinic. MUO is defined as a metastatic cancer that does not immediately have an identifiable primary site upon initial diagnosis. This is distinct from a diagnosis of CUP, which requires more extensive workup, particularly the tests recommend by the NICE guidelines in the initial diagnostic phase, which are outlined in Table 1.

To evaluate the adequacy of the referrals, specific diagnostic investigations from the NICE guidelines were selected, which were deemed essential for the initial diagnostic phase of investigation by the CUP team at the PMCC. Referrals lacking any one of these essential investigations were scored as not adequate. The essential diagnostic investigations selected were a comprehensive history and physical examination including breast, nodal areas, skin, genital, rectal, and pelvic examination, CT scans of the chest, abdomen, and pelvis, a tissue biopsy analyzed with standard histology hematoxylin and eosin, as well as immunohistochemistry (IHC). These diagnostic tests were selected as the minimum initial diagnostic criteria due to their ease of access and their necessity in confirming a diagnosis of cancer and in identifying a primary tumour with or without the presence of metastases. Other diagnostic criteria outlined in the NICE guidelines were considered to be of lower priority, not absolutely critical prior to referral to the CUP clinic.

For the analysis, each referral was scored for adequacy. The results were also quantified for each diagnostic test in order to identify how often specific diagnostics were not performed prior to referral, and by the medical specialty of the referring physician to identify if certain specialties were associated with more or less complete referrals. Descriptive statistics, including percentages and medians, were employed to detail the characteristics of patients and diseases, the diagnostic processes undertaken, and the treatments administered, without the application of formal hypothesis testing. The statistical analyses were conducted using Microsoft Excel. Referral packages were analyzed manually, with each of the criteria of the NICE guidelines tabulated and recorded as being ‘completed’ or ‘not completed’ for each referral. The criteria were considered to be ‘completed’ only if the results were available at the time of referral. All of the initial audits of the referral packages were performed by one reviewer (J.T.) using a standardized audit checklist. After the initial review, quality control was performed by a second reviewer verifying the initial audits (either I.H. or S.D.S.). In the case of a discrepancy between reviewers, the data was reviewed by all three reviewers (J.T., I.H., and S.D.S.) to ensure consistency.

## 3. Results

**Figure 1:**
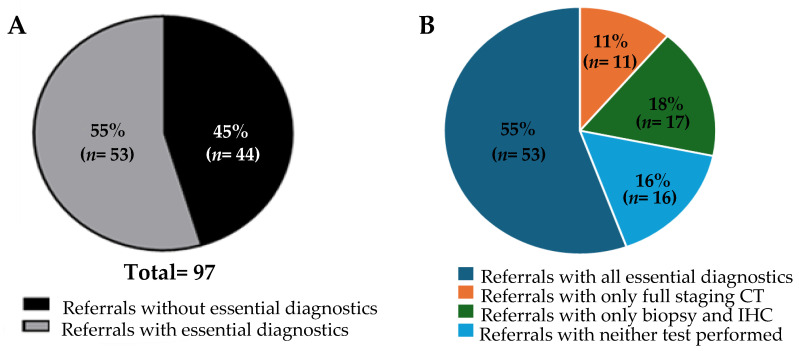
Analysis of the adequacy of referrals. (**A**) Percentage of total referrals that contained all the essential investigations to be considered adequate; (**B**) distribution of the missing essential investigations amongst the total referrals.

Our cohort consisted of referrals for 97 patients, of whom 58% (57/97) were female. The median age of the patients was 66 years. Detailed information such as presenting symptoms and number and location of metastases were not included in this analysis. As a limitation of a QI study, follow-up information such as patient outcomes are not available. Upon review, only a small number of the referrals, 7 out of 97, contained results from all of the investigations recommended by the NICE guidelines. For our analysis, however, the primary focus was to determine the adequacy of the referrals based on a reduced subset of investigations recommended by the NICE guidelines, as defined in the Methods section. When the referrals were assessed for adequacy, 55% (53/97) contained all the key investigations we deemed essential (Figure 1A). The referrals were then further reviewed to ascertain which key investigations were lacking. All of the referrals contained a sufficient history and physical examination. Therefore, the referrals were found to be inadequate due to a lack of CT scans and/or pathological examination with IHC. Indeed, we found that only CT scans were lacking in 11% of the referrals (11/97), and pathology with IHC was lacking in 18% (17/97) (Figure 1B). Neither CT scans nor biopsy with pathological assessment were performed in 16% of the total referrals (16/97). These data suggest potential barriers in the current Canadian context to obtaining these key investigations in the assessment of patients with MUO prior to referral to the CUP clinic. Likely barriers could include geographical, economic, or sociological factors, lack of resources, or lack of knowledge or training. Further studies would be required to assess the contribution of each of these factors to the results described in this article.

**Figure 2:**
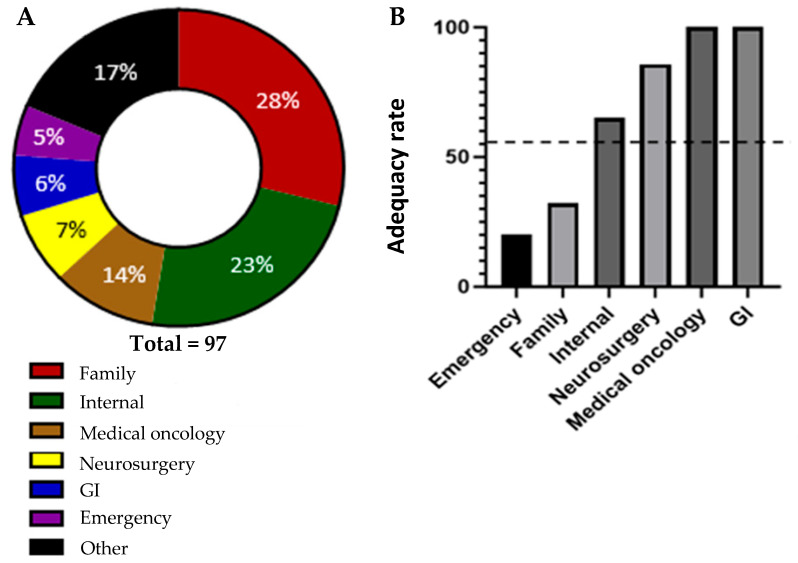
(**A**) The proportion of referrals received from different specialties and (**B**) The adequacy rate of referrals from each analyzed specialty. The dashed line represents the overall average adequacy rate (55%).

Referrals were received from a wide range of different medical disciplines. The plurality of the referrals were received from primary care physicians (family medicine), comprising a total of 28% of the referrals. The rest of the referrals came from specialists. Of those, the largest percentage of patients were referred from general internal medicine (23%). The next highest-represented referring specialties were medical oncology (14%), neurosurgery (7%), gastroenterology (GI) (6%), and emergency medicine (5%). The remaining 17% of referrals were made by a wide variety of other specialties (11 additional specialties represented by three or fewer referrals each). These findings are in keeping with the complexity of presentation of CUP, but also indicate that primary care physicians, general internists, and emergency physicians are the largest groups of medical colleagues making referrals to the CUP Clinic. There was a notable difference in the adequacy of the referrals based on referring specialty. The adequacy rate, defined as the percentage of referrals from each specialty that included all the essential diagnostics tests, varied significantly. The referring specialties (with a minimum of five referrals in this sample set) with the lowest adequacy rate were emergency medicine and family medicine, with adequacy rates of 20% and 32.1%, respectively. In contrast, the referring specialties with the highest adequacy rates were medical oncology, gastroenterology, and neurosurgery, with adequacy rates of 100%, 100%, and 85.7%, respectively (Figure 2).

## 4. Discussion

Despite attempts to standardize referrals to the CUP clinic via the publication of recommendations such as the NICE guidelines, a notable variation in the adequacy of referrals to the CUP clinic at the PMCC clearly exists (Figure 1A,B). Commonly, the clinic receives either cases that would be classified as MUO by the NICE guidelines, or instances where malignancies are not even definitively proven via histological diagnosis. This phenomenon is not unique to the PMCC and has been discussed at other CUP clinics around the world [12,13]. This variability underscores the need for a more streamlined and uniform referral system, as the current scenario reflects a wide spectrum of referral adequacy in terms of diagnostic work-up.

Only 7 out of 97 referrals that were labelled as CUP had completed all the recommended investigations outlined in the NICE guidelines. This clearly highlights a knowledge and/or availability gap in the diagnosis of CUP. Even considering the more lenient adequacy criteria we defined for this analysis, only 55% of referrals to the CUP clinic met our criteria for containing the adequate number of investigations. The heterogeneous nature of these referrals not only challenges the clinic’s operational efficiency, but also highlights the necessity for a more structured and consistent pathway for diagnosis and treatment. The broad range of referral adequacy presents an opportunity for improvement and standardization, which could significantly impact the clinical management and outcomes of patients with CUP. Notably, there was a sizeable difference in the referral adequacy rate between different specialties. This could help inform where resource allocation can be placed to yield the greatest improvements. Further investigations of other factors associated with differences in adequacy rate, such as geographic location and demographics, should be completed in order to further inform how and where improvements can best be made. Additionally, a key next step in the Knowledge-to-Action cycle is the engagement of key stakeholders to co-develop strategies to improve the referral process. This process is ongoing, but our data clearly identified that there is potentially a knowledge gap or inability to access the testing required to investigate MUO, particularly amongst primary care providers and emergency physicians (Figure 2A,B). Accordingly, as we progress through the KTA cycle, the next steps involve synthesizing the information gathered to create actionable knowledge. This synthesis will inform the development of targeted interventions aimed at enhancing the referral process. One initiative that is underway is to develop educational programmes targeted at primary care providers and emergency room physicians designed to help referring physicians understand the necessary diagnostic criteria and improve the completeness of the information provided in their referrals. This initiative will be aimed at reducing the number of inadequate referrals sent to the CUP clinic.

A more immediate intervention that has already been implemented is the launching of a Rapid Diagnostic Clinic (RDC) for solid tumours at the PMCC. This clinic is staffed by medical oncologists, but without specific specialization in CUP. All referrals sent to the CUP clinic that are deemed inadequate with the criteria used in this study are re-directed to that clinic for the timely facilitation of the investigations required. Patients that after these investigations meet the diagnosis of CUP are referred back to the CUP team or are otherwise referred to the appropriate oncologist based upon the determined site of the tumour. The creation of this RDC was a direct result of the results of the initial phase of our KTA cycle, as we identified a lack of access to biopsy as well as CT scans as a major issue with CUP referrals at our centre. From November 2024 to March 2025, the RDC has already seen 35 patients, none of whom were eventually diagnosed with CUP. Continued analysis of the impact of the RDC on patient care and ongoing engagement with clinicians making referrals to this new RDC will be required to assess if it is effective in meeting the needs of the referring physicians.

Finally, the last phase of the KTA cycle involves evaluating the impact of these interventions on clinical practice and patient outcomes. By assessing the changes in referral quality and the subsequent management of CUP cases, we can determine the effectiveness of our knowledge translation efforts. This ongoing evaluation will not only provide insights into the success of the interventions, but also highlight areas for further improvement.

By systematically following the KTA cycle, we aim to bridge the gap between existing knowledge and clinical practice, ensuring that patients with CUP receive timely and appropriate care. This structured approach to knowledge translation will contribute to the overall enhancement of healthcare delivery and outcomes in the management of complex cancer cases.

## 5. Conclusions

Based on the analyzed data, approximately 45% of referrals to the CUP clinic at the PMCC did not adequately complete the minimum diagnostic criteria to determine that the specialized knowledge of a CUP clinic would be necessary or valuable. This large percentage of referrals increases the resource drain on a small, specialized team that diminishes the resources available for patients with CUP that require this specialized knowledge. This presents an opportunity for the improvement and standardization of early diagnostics and referrals, which could significantly impact the clinical management and outcomes of patients with CUP. Obviously, this study has the inherent limitations of being retrospective in nature and from a single centre with a relatively small sample size. That being said, however, this study has led to changes in practice that have impacted patient care at our centre, which is the final goal of the KTA cycle.

## Figures and Tables

**Table 1:** NICE-recommended investigations for the initial phase of diagnosis for CUP [6].

Investigation:
Comprehensive history and physical examination including breast, nodal areas, skin, genital, rectal, and pelvic examination
Full blood count; urea, electrolytes, and creatinine; liver function tests; calcium; urinalysis; lactate dehydrogenase
Chest X-ray
Myeloma screen (when there are isolated or multiple lytic bone lesions)
Symptom-directed endoscopy
Computed tomography (CT) scan of the chest, abdomen, and pelvis
Prostate-specific antigen (PSA) in men
Cancer antigen 125 (CA125) in women with peritoneal malignancy or ascites
Alpha-fetoprotein (AFP) and human chorionic gonadotrophin (hCG) (particularly in the presence of midline nodal disease)
Testicular ultrasound in men with presentations compatible with germ-cell tumours
Biopsy and standard histological examination, with immunohistochemistry where necessary, to distinguish carcinoma from other malignant diagnoses

## Data Availability

The data presented in this study are available on request from the corresponding author due to patient confidentiality.
